# Clinical management and prevention of dental caries in athletes: A four-year randomized controlled clinical trial

**DOI:** 10.1038/s41598-018-34777-x

**Published:** 2018-11-19

**Authors:** C. Frese, T. Wohlrab, L. Sheng, M. Kieser, J. Krisam, F. Frese, D. Wolff

**Affiliations:** 1 Department of Conservative Dentistry, School of Dental Medicine, University Hospital, Heidelberg, Germany; 2 Institute of Medical Biometry and Informatics, Ruprecht Karls University, Heidelberg, Germany; 3 Department of Conservative Dentistry, School of Dental Medicine, University Hospital Tübingen, Tübingen, Germany; 4 Clinic St. Elisabeth, Private Practice for Sports Medicine, Heidelberg, Germany

## Abstract

The aims of this four-year randomized controlled clinical trial were to gain insights into management and prevention of dental caries and the effect of stannous fluoride products in athletes. Fifty-four participants were randomized into test and control groups. The test group used special stannous fluoride products. The primary endpoint dental caries was assessed by the ICDAS-II-System and analyzed both by a linear mixed model for repeated measures and a generalized linear mixed model. During the observation period an increase in caries-free surfaces from 64.91 ± 6.42 at baseline to 73.22 ± 4.43 was observed. In surfaces with caries superficialis and caries media, a decrease from 13.94 ± 5.70 and 2.96 ± 2.55 surfaces at baseline to 7.89 ± 3.18 and 0.46 ± 0.78 after 2.5 years was noted, respectively. The analysis showed no effect of stannous fluoride products, but a significant difference for the time of examination (p < 0.0001). In addition, it could be shown that at any time of examination, the odds of developing caries media on a new surface was significantly lower than at baseline (up to 25-times). Due to biannual dental examinations, professional tooth cleaning and restorative treatment the number of caries-free surfaces increased and the odds of a new surface to be afflicted with caries media decreased 25-fold.

## Introduction

In recent years there has been an increasing interest in the oral health of elite and amateur athletes. Although the research in this field is limited, there is a general consensus that oral health of athletes is poor and can be compared to non-athletes with low socio-economic status^1,2^. The main dental problems of the athletes are caries (15–75%), dental erosion (36–85%), trauma (14–47%), periodontal problems (15%) and pericoronitis/impacted third molars. Furthermore, in some studies, a negative impact of poor oral health or trauma on sports performance was reported^3^. This impact on performance might arise from pain, systemic inflammation due to active periodontitis^4^, and/or reduced confidence because of an impaired oral status^5^. The crucial factors that influence the oral health of athletes are nutritional factors, host regulation and behavioural characteristics^1,2^. Training and exercise are often related to frequent consumption of high carbohydrate-containing sports nutrition or sports drinks^6,7^. High carbohydrate intake promotes the development of carious lesions, whereas acidic sports drinks with low pH contribute to the development of dental erosion^8–10^. A fluid and electrolyte deficit during exercise, due to water and sweat loss, may affect exercise performance, reduces salivary flow and induces dehydration and a dry mouth^11,12^. In this case, the negative effect of high carbohydrate-containing sports nutrition or sports drinks on oral health is assumed to increase manifold.

In contrast to complex and time-consuming therapeutic interventions, prevention of dental caries can be achieved easily and cost-effectively. In caries prevention, topical application of fluoride compounds is considered to be the most important preventive agent. On the one hand, fluoride ions can be partly adsorbed and incorporated into the crystal surface of the enamel and offer direct protection against demineralization of the tooth hard tissue^13^. On the other hand, in contact with calcium-containing saliva and the tooth surface, fluorides can form a calcium-fluoride precipitate on the tooth surface. These calcium-fluoride deposits are capable of reducing the surface micro-hardness of enamel, and thus counteracting the carious process^13,14^. With regard to the prevention of erosive wear due to acidic food or beverages, it is known that the combination of tin and fluoride shows an even higher efficacy in comparison to tin or fluoride used alone^15,16^. Stannous fluoride reacts with hydroxyapatite to form CaF_2_, Sn_2_OHPO_4_, Sn_3_F_3_PO_4_, and Ca(SnF_3_)_2_^17^. The tin ions precipitate at the tooth surface and within the acquired enamel pellicle to form a protective layer, which is more acid-resistant than pure CaF_2_^18,19^.

Thus, according to the Patient, Intervention, Comparison, Outcome format (PICO) the aim of this randomized controlled clinical trial (RCT) was to first gain insights into the clinical management and prevention of dental caries in a population of athletes. The intervention of the RCT includes: i) A randomized daily usage of test products: special stannous fluoride toothpaste/mouth rinse and ii) half-year follow-up appointments for all participants; and professional tooth cleaning and oral hygiene instructions for all participants. The control group used their conventional fluoride-containing oral hygiene products. Related to the outcomes it was hypothesized that structured biannual dental visits in combination with professional tooth cleaning and oral hygiene instructions might affect caries experience. Furthermore, it was hypothesized that the use of special stannous fluoride toothpaste/mouth rinse might have an impact on caries development. Based on the data, the aim was to give recommendations for caries disease management in sports medicine and dentistry.

## Results

### General Data

Participants were recruited between March and October 2013. The baseline examination was performed in October 2013. Follow-up appointments were carried out in April and October of the years 2014–2017.

Of the 54 athletes, 41 were male and 13 were female. The mean age of the athletes was 36.53 ± 9.49 years (range 20–60 years). To compare the similarities between the test and control group according to the inclusion criteria, general data and nutritional habits are depicted in Table 1.

**Table 1:** Descriptive analysis of general data according to the inclusion criteria of test and control group depicted in mean values, standard deviation and median. Comparisons for test and control group were carried out in order to exclude specific differences at baseline (Mann-Whitney-U-test for continuous, and chi-squared test for categorical variables).

Variable	Control group n = 27	Test group n = 27	Total n = 54	p-value*
**Age**				
- Mean+/− SD	36.26 +/− 8,68	34.44 +/− 10,01	35.35 +/− 9,32	0.387
- Median	36	34	35,5	
**Gender**				
- Male	24 (88.9%)	17 (63.0%)	41 (75.9%)	0.026
- Female	3 (11.1%)	10 (37.0%)	13 (24.1%)	
**BMI [kg/m²]**				
- Mean +/− SD	22.98 +/− 2.23	22.91 +/− 2.79	22.95 +/− 2.50	0.729
- Median	22.96	22.34	22.81	
**Discipline**				
- Triathlon	15 (55.6%)	17 (63.0%)	32 (59.3%)	0.254
- Running	2 (7.4%)	6 (22.2%)	8 (14.8%)	
- Cycling	3 (11.1%)	1 (3.7%)	4 (7.4%)	
- Rowing	0 (0.0%)	1 (3.7%)	1 (1.9%)	
- Running + cycling	4 (14.8%)	2 (7.4%)	6 (11.1%)	
- Cycling + swimming	1 (3.7%)	0 (0.0%)	1 (1.9%)	
- Running + swimmimg	2 (7.4%)	0 (0.0%)	2 (3.7%)	
**Training [h/week]**				
- Mean +/− SD	9.07 +/− 3.83	9.24 +/− 3.48	9.16 +/− 3.63	0.735
- Median	8	9	8	
**Beverage during excercise**				
- Water	16 (59,3%)	12 (44,4%)	28 (51,9%)	0.376
- Sports drinks	2 (7,4%)	4 (14,8%)	6 (11,1%)	
- Juice	0 (0,0%)	1 (3,7%)	1 (1,9%)	
- Water + sports drinks	4 (14,8%)	7 (25,9%)	11 (20,4%)	
- Water + juice	5 (18,5%)	2 (7,4%)	7 (13,0%)	
- Sports drinks + juice	0 (0,0%)	1 (3,7%)	1 (1,9%)	
**Nutrition during excercise**				
- None	8 (29.6%)	8 (29.6%)	16 (29.6%)	0.907
- Bars/Gels	12 (44.4%)	14 (51.9%)	26 (48.1%)	
- Fruit	6 (22.2%)	4 (14.8%)	10 (18.5%)	
- Sandwiches	1 (3.7%)	1 (3.7%)	2 (3.7%)	

^*^p-value based on the Mann-Whitney-U-test for continuous, and chi-squared test for categorical variables.

**Figure 1:**
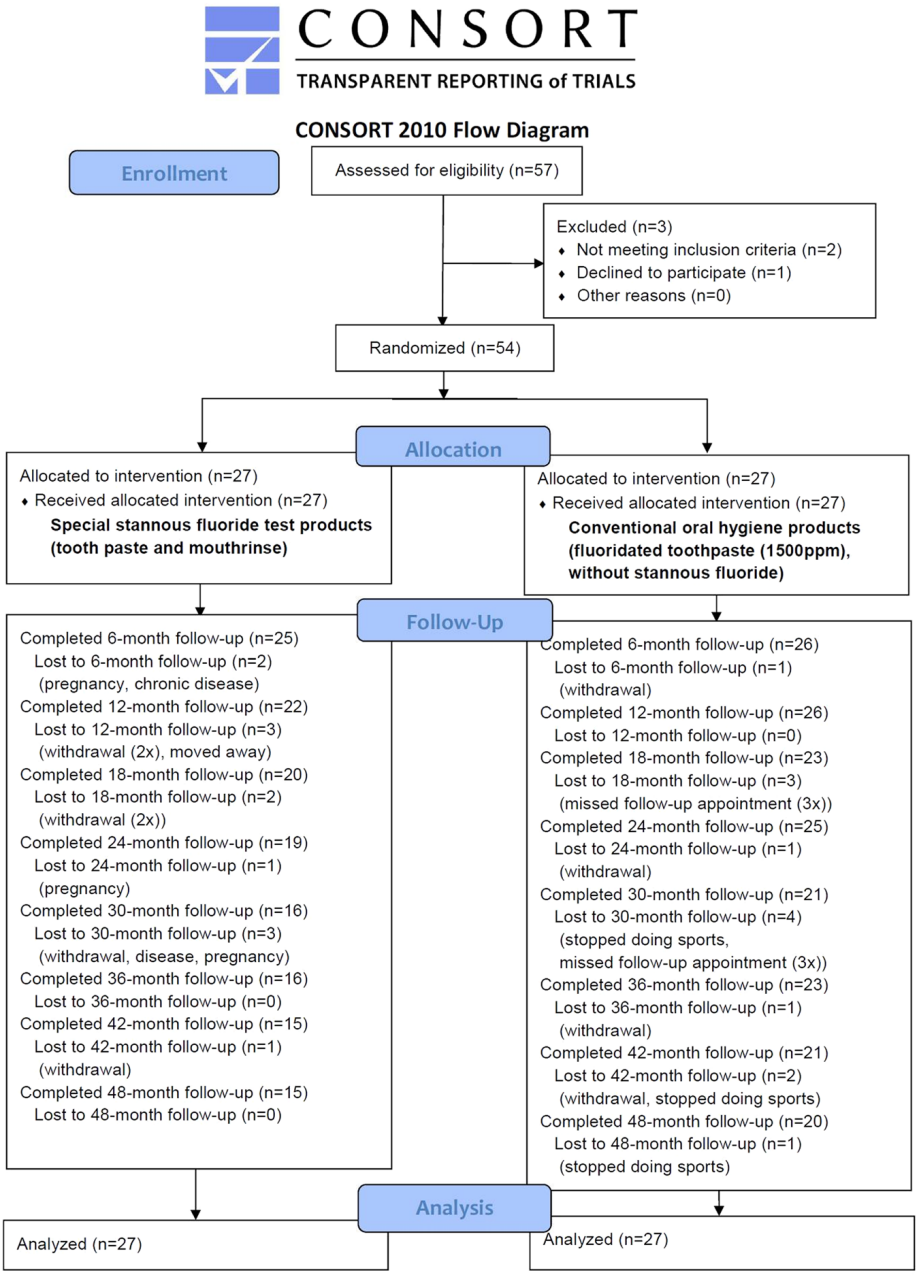
Depicts the CONSORT flow diagram, the adverse events as well as the reasons for withdrawal. Data were analysed according to the “Intention-to-treat analysis”. With 19 patients withdrawing from the study, the overall dropout rate was 35.19% with 7 subjects resigning from the control, and 12 subjects from the test group. Six control subjects missed one follow-up appointment each, but continued to participate in the remaining follow-up examinations afterwards.

Table 2 shows the number of teeth, the number of missing teeth, the surfaces with direct and indirect restorations (ICDAS F_3+4+6_) as well as the caries prevalence at baseline. Of the altogether 54 test subjects at baseline, 29 (53.70%) participated in all follow-up examinations. With 19 patients withdrawing from the study, the overall dropout rate was 35.19% with 7 subjects resigning from the control, and 12 subjects from the test group. Figure 1 depicts the CONSORT flow diagram, the adverse events as well as the reasons for withdrawal (16). Six control subjects missed one follow-up appointment each, but continued to participate in the remaining follow-up examinations afterwards.

**Table 2:** Descriptive analysis of the caries-free surfaces and the surfaces with caries superficialis (D_1+2_), caries media (D_3+4_), and caries profunda (D_5+6_), number of teeth, number of missing teeth and restored surfaces (tooth colored restoration/amalgam F_3+4_ and crowns F_6_) at baseline.

Variable	Control group n = 27	Test group n = 27	Total n = 54	p-value*
**Number of teeth**				
- Mean +/− SD	27.37 +/− 1.15	27.26 +/− 1.46	27.31 +/− 1.30	0.626
- Median	28	28	28	
- Min, Max	24, 28	24, 28	24, 28	
**Number of missing teeth**				
- Mean +/− SD	0.19 +/− 0.48	0.26 +/− 0.86	0.22. +/− 0.69	0.767
- Median	0	0	0	
- Min, Max	0, 2	0, 4	0, 4	
**Restored surfaces with tooth colored restoration/ amalgam (F**_**3+4**_ **)**				
- Mean +/− SD	9.37 +/− 6.31	6.81 +/− 5.51	8.09 +/− 6.00	0.170
- Median	9	6	6	
- Min, Max	0, 20	0, 22	0, 22	
**Restored surfaces with crowns (F**_**6**_ **)**				
- Mean +/− SD	7.19 +/− 11.06	2.89 +/− 5.24	5.04 +/− 9.11	0.241
- Median	0	0	0	
- Min, Max	0, 38	0, 16	0, 38	
**caries-free surfaces (D**_**0**_ **)**				
- Mean +/− SD	65.59 +/− 5.83	64.22 +/− 7.01	64.91 +/− 6.42	0.405
- Median	66	63	64	
- Min, Max	55, 79	48, 80	48, 80	
**surfaces with caries (D**_**1–6**_ **)**				
- Mean +/− SD	16.52 +/− 4.97	17.56 +/− 7.10	17.04 +/− 6.09	0.742
- Median	15	18	16	
- Min, Max	5, 25	4, 31	4, 31	
**Caries superficialis (D**_**1+2**_ **)**				
- Mean +/− SD	13.33 +/− 5.00	14.56 +/− 6.36	13.94 +/− 5.70	0.400
- Median	13	15	13.5	
- Min, Max	5, 25	3, 27	3, 27	
**Caries media (D**_**3+4**_ **)**				
- Mean +/− SD	3.00 +/− 3.31	2.93 +/− 1.54	2.96 +/− 2.55	0.361
- Median	2	3	3	
- Min, Max	0, 16	0, 6	0, 16	
**Caries profunda (D**_**5+6**_ **)**				
- Mean +/− SD	0.19 +/− 0.56	0.07 +/− 0.27	0.13 +/− 0.44	0.606
- Median	0	0	0	
- Min, Max	0, 2	0, 1	0, 2	

*p-value based on the Mann-Whitney-U-test.

### Caries prevalence at baseline

The number of caries-free surfaces (ICDAS D_0_), surfaces with caries superficialis (ICDAS D_1+2_), surfaces with caries media (ICDAS D_3+4_), and surfaces with caries profunda (ICDAS D_5+6_) at baseline are shown in Table 2. At baseline, both the test and control group showed similar caries prevalence, the mean of decayed surfaces (ICDAS D_1–6_) being 17.04 +/− 6.09 (p = 0.742).

Table 3 shows the caries-free surfaces, the surfaces with caries superficialis (D_1+2_), caries media (D_3+4_), and caries profunda (D_5+6_), number of teeth, number of missing teeth and restored surfaces (tooth colored restoration/amalgam F_3+4_ and crowns F_6_) after four years.

**Table 3:** Descriptive analysis of the caries-free surfaces and the surfaces with caries superficialis (D_1+2_), caries media (D_3+4_), and caries profunda (D_5+6_), number of teeth, number of missing teeth and restored surfaces after four years.

Variable	Control group n = 20	Test group n = 15	Total n = 35	p-value*
**Number of teeth**				
- Mean +/− SD	27.15 +/− 1.35	27.13 +/− 1.81	27.14 +/− 1.54	0.580
- Median	28	28	28	
- Min, Max	24, 28	22, 28	22, 28	
**Number of missing teeth**				
- Mean +/− SD	0.15 +/− 0.37	0.47 +/− 1.55	0.29 +/− 1.05	0.978
- Median	0	0	0	
- Min, Max	0, 1	0, 6	0, 6	
**Restored surfaces with tooth colored restoration/amalgam**				
- Mean +/− SD	9.20 +/− 6.28	6.20 +/− 7.02	7.91 +/− 6.68	0.120
- Median	9.5	3	5	
- Min, Max	0, 20	0, 22	0, 22	
**Restored surfaces with crowns**				
- Mean +/− SD	6.80 +/− 10.93	4.27 +/− 5.68	5.71 +/− 9.04	0.792
- Median	1.5	2	2	
- Min, Max	0, 38	0, 17	0, 38	
**caries-free surfaces (D**_**0**_ **)**				
- Mean +/− SD	73.15 +/− 7.01	72.00 +/− 4.57	72.66 +/− 6.03	0.300
- Median	74.5	72	74	
- Min, Max	57, 81	62, 79	57, 81	
**surfaces with caries (D**_**1–6**_ **)**				
- Mean +/− SD	8.30 +/− 4.92	9.40 +/− 3.66	8.77 +/− 4.40	0.291
- Median	8	9	9	
- Min, Max	3, 20	4, 15	3, 20	
**Caries superficialis (D**_**1+2**_ **)**				
- Mean +/− SD	7.65 +/− 4.61	9.00 +/− 3.70	8.23 +/− 4.24	0.192
- Median	6.5	9	7	
- Min, Max	3, 19	4, 15	3, 19	
**Caries media (D**_**3+4**_ **)**				
- Mean +/− SD	0.50 +/− 0.83	0.40 +/− 0.74	0.46 +/− 0.78	0.684
- Median	0	0	0	
- Min, Max	0, 3	0, 2	0, 3	
**Caries profunda (D**_**5+6**_ **)**				
- Mean +/− SD	0.15 +/− 0.67	0.00 +/− 0.00	0.09 +/− 0.51	0.419
- Median	0	0	0	
- Min, Max	0, 3	0, 0	0, 3	

^*^p-value based on the Mann-Whitney-U-test.

### Development of caries-free surfaces (ICDAS D0)

**Figure 2:**
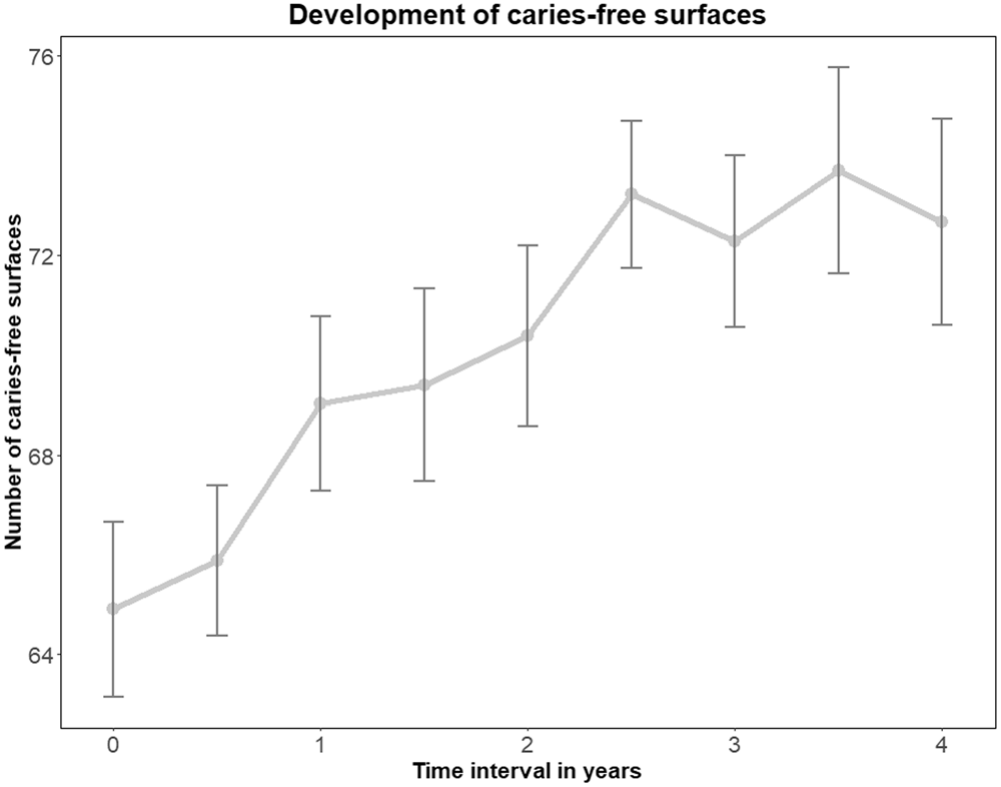
Increase of caries-free surfaces over the course of the four-year observation period with mean values and standard deviation. The increase of caries-free surfaces has to be interpreted in the context of restorative treatments as the scoring of the tooth surface switches from an unrestored (F_0_) and decayed surface (D_3,4,5 or 6_) to a restored (F_3,4, or 6_) and caries-free surface (D_0_).

Figure 2 shows the development of caries-free surfaces over the four-year observation period.

At baseline the mean of caries-free surfaces of the participants was 64.91 +/− 6.42. During the observation period an increase in caries-free surfaces was observed up to the 2.5 year recall (t = 2.5) showing 73.22 +/− 4.43 caries-free surfaces. After two and a half years, the number of caries-free surfaces remained constant, and the mean of caries-free surfaces recorded at the four-year recall (t = 4.0) was 72.66 +/− 6.03.

The analysis by the linear mixed models for repeated measures (MMRM) showed a significant difference for the time of examination (p < 0.0001, Table 4). Group-specific differences were not observed, as the intervention with special stannous fluoride-containing oral hygiene products did not show a statistical significant effect on the development of caries-free surfaces (p = 0.8687, Table 4).

**Table 4:** Results of the linear mixed models for repeated measures (MMRM) for caries-free surfaces and surfaces with caries superficialis.

	Caries-free surfaces Standard			Caries superficialis Standard		
	Estimate	error	p-value	Estimate	error	p-value
Group (intervention SnCl_2_)			0.8687			0.5669
Time of examination			**<0.0001			**<0.0001
Group*time of examination			0.2316			0.2226
Age	−0.0125	0.0784	0.8741	−0.0630	0.0516	0.2296

### Development of carious surfaces: Caries superficialis (ICDAS D_1+2_)

**Figure 3:**
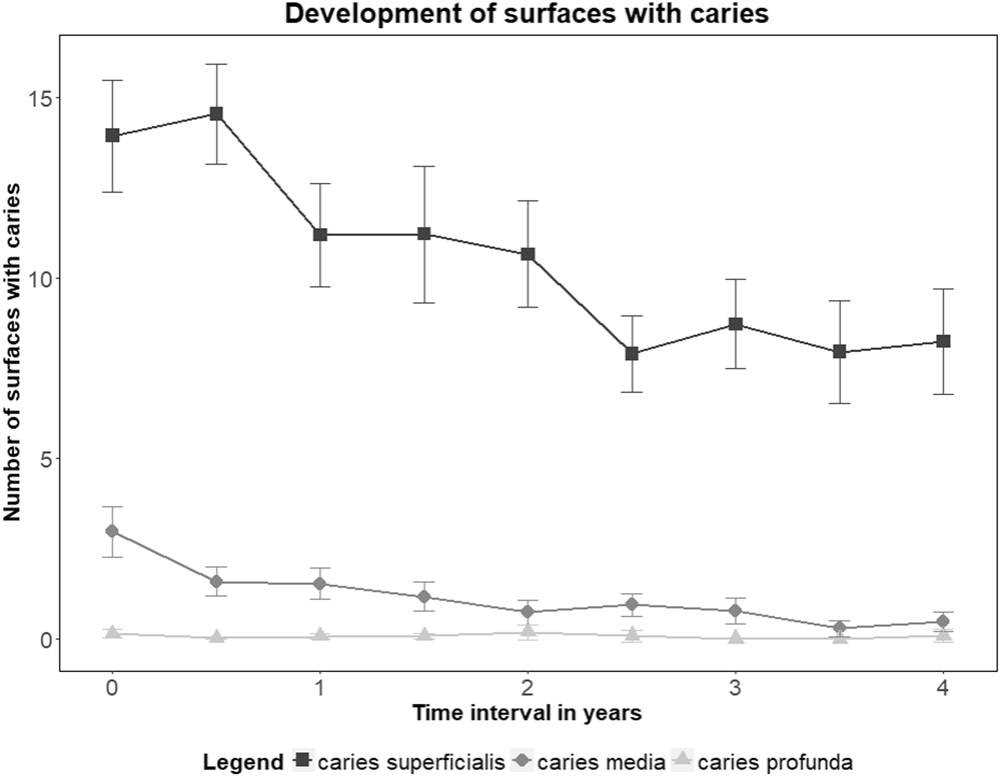
Decrease of surfaces with caries superficialis (D_1+2_), caries media (D_3+4_) and caries profunda (D_5+6_) over the course of the four-year observation period with mean values and standard deviation.

Figure 3 shows the development of carious surfaces over the four-year observation period divided into caries superficialis (ICDAS D_1+2_), caries media (ICDAS D_3+4_), and caries profunda (ICDAS D_5+6_). At baseline the surfaces with caries superficialis of the participants showed a mean of 13.94 +/− 5.70. During the observation period, a decrease in surfaces with caries superficialis was observed until the 2.5 year recall (t = 2.5) showing on average 7.89 +/− 3.18 surfaces with caries superficialis. After two and a half years, the number of surfaces with caries superficialis remained constant; at the four-year recall (t = 4.0), the number of surfaces with caries superficialis showed a mean of 8.23 +/− 4.24.

The analysis by the linear mixed model for repeated measures (MMRM) showed a significant difference for the time of examination (p < 0.0001, Table 3). Group-specific differences were not observed, as the intervention with special stannous fluoride-containing oral hygiene products did not show a statistically significant effect on the development of surfaces with caries superficialis (p = 0.5669, Table 4).

### Development of carious surface: Caries media (ICDAS D_3+4)_

With regard to caries media (ICDAS D_3+4_) lower prevalence was recorded in comparison to caries superficialis (Fig. 3). At baseline the surfaces with caries media of the participants showed a mean of 2.96 +/− 2.55. Due to subsequent restorative treatment of surfaces with caries media, their number decreased over the four-year observation period. After two years the surfaces with caries media showed a mean of 0.73 +/− 1.13, and after the four-year observation period a mean of 0.46 +/− 0.78 (Table 3).

Analysis by the generalized linear mixed model (GLMM) showed a significant difference for the time of examination (p < 0.0001). In addition, the odds ratios for the time of examination were calculated and it could be shown that at any time of examination, the odds of developing a new surface with caries media was significantly lower than at baseline (Table 5). Associated with periodic dental examinations, the odds of developing a new surface with caries media was four times lower after six months, 17x lower after two years, and 25x lower after four years (Table 5).

**Table 5:** Odds ratios and 95% confidence interval for the increasingly lowered risk (1/Odds ratio) of developing a new surface with caries media for every time of examination in comparison to baseline.

Time of examination [y]	Odds ratio	95% CI		1/Odds ratio	95% CI	
0.5	0.247	0.078	0.783	4.049	1.277	12.821
1	0.172	0.061	0.487	5.814	2.053	16.393
1.5	0.107	0.034	0.340	9.346	2.941	29.412
2	0.058	0.019	0.174	17.241	5.747	52.632
2.5	0.124	0.038	0.403	8.065	2.481	26.316
3	0.062	0.020	0.196	16.129	5.102	50.000
3.5	0.016	0.004	0.060	62.500	16.667	250.000
4	0.039	0.012	0.123	25.641	8.130	83.333

### Development of carious surfaces: Caries profunda (ICDAS D_5+6_)

Deep and cavitated carious lesions, caries profunda (D_5+6_), were only recorded in individual participants (Fig. 3) and restorative treatment was subsequently carried out. The mean number of surfaces was low throughout the four-year observation period and variations could not be observed. Therefore, only a descriptive analysis can be provided, since the GLMM did not converge. After three (t = 3.0) and three and a half years (t = 3.5) no caries profunda was recorded. The maximum value of surfaces with caries profunda recorded in this study was n = 4 after two years of examination (t = 2.0). At any other time points, the number of surfaces with caries profunda was n = 1.

## Discussion

This four-year randomized controlled clinical trial is the first investigation that gives insight into management strategies for the prevention of caries in athletes over a four-year period. To the best of our knowledge, no comparable data on longitudinal observations in caries management exist in the literature so far^2^.

With special regard to caries prevalence, we used the ICDAS-II score which allows for detection of different stages of carious decay: no decay, superficial decay, medium decay and deep lesions. Therefore, where preventive care is indicated, a distinction can be made between lesions that require restorative treatment and lesions that are located in the enamel without cavitation^20,21^. Also the reversal of initial carious lesions due to remineralization of the enamel is detectable as we could see a decrease in surfaces with caries superficialis from 13.94 +/− 5.70 at baseline to 8.23 +/− 4.24 after four years. The remarkable increase of caries-free surfaces has to be interpreted in the context of restorative treatments as the scoring of the tooth surface switches from an unrestored (F_0_) and decayed surface (D_3,4,5 or 6_) to a restored (F_3,4, or 6_) and caries-free surface (D_0_). Nevertheless, the ICDAS II-Score is a valid and reliable tool to record and monitor carious lesions at different stages^22,23^, and therefore is the right tool for data assessment in longitudinal clinical studies that focus on caries development^21^.

In this study, it could be shown that the time of examination had significant influence on the increase of sound enamel surfaces and on the decrease of surfaces with carious lesions (p < 0.0001, Tables 3 and 4). Up to 8.9 +/− 1.05 additional caries-free surfaces were recorded after three and a half years of observation (t = 3.5). Regular dental visits in combination with professional tooth cleaning and oral hygiene instruction seem to have a beneficial effect on caries management in the population investigated here. However, in this population and under the conditions of this trial the special stannous-fluoride test products did not show an additional effect on caries development (Table 4). The athletes in this investigation belong to a group with a high awareness of (oral) health and represent a group with high socio-economic status. The additional effect of stannous fluoride might have been visible if the participants of this study would have been chosen from a social deprived population with a high caries risk. Compared to the age group of 35–44 years of the German Study on Oral Health conducted in 2016 it was obvious that a low socio-economic status is associated with a 2.2 points higher DMFT-index.

The significant increase in caries-free surfaces and reduction in surfaces with caries media and caries profunda was due to restorative treatment of carious surfaces. All participants with carious lesions requiring treatment (surfaces with ICDAS scores D_3,_ D_4_, D_5_ and D_6_) were scheduled for treatment appointments in our department, or they consulted their family dentist. The results show that surfaces with caries media could be reduced significantly (Fig. 3). Additionally, it could be shown that at any time of examination, the odds of developing a new surface with caries media was significantly lower that at baseline (Table 5). Associated with periodic dental examinations, the risk of developing a new surface with caries media was four times lower after six months, 17x lower after two years and 25x lower after four years (Table 5). This leads to the conclusion that regular dental care for athletes reduces the caries risk of the individual. When comparing the results of caries prevalence in the athletes from this longitudinal study with the German Study on Oral Health conducted in 2016, the caries prevalence of athletes after the four-year period were distinctly lower than in the German population (DMFT-values) (Jordan and Micheelis, 2016).

Another known risk factor for caries is the frequent consumption of carbohydrate-containing sports nutrition to maintain athletic performance^11,24^. At baseline, the nutritional habits during training were not significant different between the two groups (Table 1). Over the observation period, a significant reduction of carious surfaces was observed in all participants, as explained above. The participants, who strictly adhered to the repetitive dental visits in this RCT, acquired an increased awareness of oral health related to therapeutic interventions, effective oral hygiene and/or dietary interventions^25^. As a further strategy to keep the number of surfaces with carious decay low, regular biannual follow-up appointments with professional tooth cleaning sessions and oral hygiene instructions should be recommended for athletes.

Drawing conclusions from these findings, we became aware of the following weaknesses and limitations of the study: i) The number of test subjects and controls that were included does not allow to draw confirmatory conclusions from the data; ii) in RCTs evaluating oral hygiene formulations over years, the choice of controls is problematic, because a placebo or negative control (here fluoride-free toothpaste) is unethical, and therefore not possible and iii) between the two groups the loss to follow-up is unequal over the four years of investigation. With 19 patients withdrawing from the study, the overall dropout rate was 35.19% with 7 subjects resigning from the control, and 12 subjects from the test group. Six control subjects missed one follow-up appointment each, but continued to participate in the remaining follow-up examinations afterwards (Fig. 1).

Despite all limitations, we tried to provide a structured prevention regimen and regular follow-up appointments for all participants, in order to raise awareness of oral health. However, there were many reasons to drop-out. Some of the participants, being university students, moved away or were unwilling to participate any longer, two women got pregnant (both test group) and one participant suffered from a chronic disease and was not able to participate any longer (test group). Furthermore, limitations of clinical monitoring using the ICDAS score were the proximal surfaces of the teeth. First, in contact with neighboring teeth a sufficient and valid monitoring was not possible. Second high difficulties in obtaining ethical approval for taking systematically radiographs were existent and therefore a longitudinal monitoring of approximal surfaces was not included in the study. To address these shortcomings, further clinical research and, if possible, greater cohorts of participants would be preferable to support the data obtained in this study.

It can be concluded that caries-free surfaces increased, due to regular biannual dental examinations with restorative treatment, professional tooth cleaning and oral hygiene instructions, and the odds of developing a new surface with caries media decreased to the factor 25 after four years. Therefore, management and prevention of caries in athletes might be realized and can be summarized as follows: i) periodic scheduling of athletes: 6-months intervals; ii) professional tooth cleaning and oral hygiene instructions; iii) general recommendation of fluoride-containing oral hygiene products.

## Methods

### Study design

This randomized controlled clinical trial meeting current ethical standards^26^ was conducted after obtaining approval from the local medical ethics committee of the Medical Faculty of the University of Heidelberg (S-566/2012) and was performed in accordance with relevant guidelines (e.g. the CONSORT statement)^27^. The study was registered at the German Clinical Trials Registry Platform (DRKS00005019; date of registration 2013/05/27) that is linked to the International Clinical Trials Registry Platform of the World Health Organization (WHO). This trial was conducted as a randomized controlled clinical trial covering an observation period of four years with one baseline examination and eight biannual follow-up examinations, respectively.

Fifty-four athletes from sports clubs or the university, who perform five or more hours of endurance training per week, participated in this investigation and gave written informed consent. The study was officially announced in the sports clubs and at the university, and athletes that met the inclusion criteria and were willing to participate were invited for study appointments. The participants were randomized by block randomization (sequentially numbered envelopes) into either test or control group, as is depicted in the CONSORT flow diagram (Fig. 1)^27^. The random allocation sequence was generated by the statistician; enrolment and assignment of participants to interventions was done by the principal investigator. The participants in the test group were instructed to use a special stannous fluoride-containing [(AmF)/NaF/SnCl_2_] mouth rinse (500 ppm F^-^, 800 ppm Sn^2+^), 1 × 30 s per day and a special toothpaste containing NaF/Sn(2^+^) and the biopolymer chitosan (elmex EROSIONSSCHUTZ, CPGABA GmbH, Hamburg, Germany) for daily oral hygiene at home^28,29^.

The participants in the control group did not get any products with the exception of the instruction to use fluoridated toothpaste (1500 ppm) with their conventional oral hygiene products at home. They were explicitly told not to use stannous fluoride containing oral hygiene products.

### Inclusion and exclusion criteria

The inclusion criteria were: participants who i) were older than 18 years, ii) gave written informed consent, iii) declared that they performed endurance sports (running, cycling, swimming, track and field, triathlon) with a cumulative weekly training time of five or more hours, and iv) were in good general health and not restricted in practicing oral hygiene.

The exclusion criteria were: participants who (i) were under the age of 18, ii) gave no written informed consent, iii) performed a cumulative weekly training of less than five hours, iv) were restricted in practicing oral hygiene, v) were pregnant or nursing, vi) had been or were still taking part in another clinical study within the last 30 days, vii) took antibiotics within the last 30 days, viii) were dental students or dental staff members.

### Clinical oral investigation

One blinded calibrated examiner (TW) performed the standardized clinical examination every six months. The study protocol was comprised of anamnesis in written form, intraoral inspection, assessment of caries index (ICDAS II), standardized photographs and a professional tooth cleaning session including oral hygiene instructions. The aim of this preventive setting was to generate an increased awareness of oral health related to therapeutic interventions, effective oral hygiene and/or dietary interventions. Intraoral examination was done after professional tooth cleaning using a dental operating light, binocular loupes (magnification 2.5x), plain mirrors, and diagnostic probes. Calibration was done using the ICDAS e-learning and online calibration tool.

### Assessment of caries prevalence

For assessment of caries prevalence, the International Caries Detection and Assessment System II (ICDAS II) was applied on three sites per tooth (buccal, oral, occlusal/incisal)^30^. In individual cases when valid medical indications were seen, bitewing radiographs were taken. However due to the inhomogeneity of this data, it could not be considered in the statistical analysis. ICDAS II values were obtained at every recall appointment, and depending on the number of teeth, a maximum of 84 surfaces could be recorded per participant. Using the ICDAS criteria, the sites were recorded by a 0 to 6 scoring system: 0 = sound, *no caries*; 1 = first visible sign of non-cavitated caries after 5 sec air-drying, *caries superficialis*; 2 = first visible sign of non-cavitated caries when tooth is wet and dry, *caries superficialis*; 3 = microcavitation in enamel, *caries media*; 4 = caries lesion extending into dentine (underlying shadow), *caries media*; 5 = small cavitated lesion, and dentine is visible in <50% of the surface, *caries profunda*; 6 = large cavitated lesions, and dentine is visible >50% of the surface, *caries profunda*. If carious lesions requiring treatment were observed (ICDAS codes D_3–6_), subsequent caries removal and restorative treatment was carried out.

### Statistical analysis

This study, being a RCT with a four-year observation period, had to be prepared for exclusion and Nstudy withdrawal, and the subsequent loss of test persons’ data. The number of subjects resigning from the study, as well as their reasons for doing so, were documented in the CONSORT flow diagram (Fig. 1). At the end of the trial all 54 patients were analyzed according to the intention-to-treat principle (ITT), i.e. all randomized patients were included regardless of any protocol violations and analyzed according to the group they were allocated to originally. Hence, also those patients who dropped out during the trial or were lost to follow-up were included in the analyses.

The data were analyzed using descriptive statistics, evaluating mean, standard deviation, minimum, median and maximum for all baseline and follow-up parameters.

The statistical analysis of the ICDAS II system with regard to the carious decay of athletes was assessed by calculating cumulative D-S_1–6_ indices combining the cut-off points: D_0_-S (ICDAS code 0), D_1+2_-S (ICDAS codes 1 and 2 representing *caries superficialis*), D_3+4_-S (ICDAS codes 3 and 4, representing *caries media*) and D_5+6_-S (ICDAS codes 5 and 6, representing *caries profunda*). The primary endpoint dental caries for the ICDAS codes D_0_ and D_1+2_ was analyzed by a linear mixed model for repeated measures (MMRM), which allowed us to include patients with an incomplete follow-up into the analysis. Due to the non-continuous character of the ICDAS codes D_3+4_ and D_5+6_ a generalized linear mixed model (GLMM) with the dependent variable “number of surfaces with caries media/profunda >0” (yes/no) was used. Effect estimates for comparing each time point between groups were calculated by Least Squares MEANS (LSMEANS) statements together with the 95% confidence intervals and descriptive p-values.

To compare the time points of investigation, odds ratios were calculated by the use of LSMEANS. Further pairwise comparisons between the test and control group were made using U-test for continuous and ordinal data, while categorical data were compared using the χ^2^-test. All analyses were done using the software package SAS^®^ System 9.4 (SAS Inc., Cary/NC, USA), or higher. RStudio Desktop 1.1.383 was used to create the graphics.

## Electronic supplementary material

CONSORT Check list

Clinical trial protocol

## Data Availability

The authors state that with special regard to the availability of the data of this study there are no restrictions or third party interests to declare.
