# Supplementary material for: Clinical management and prevention of dental caries in athletes: A four-year randomized controlled clinical trial
Source: Sci Rep. 2018 Nov 19;8:16991. doi: 10.1038/s41598-018-34777-x (PMC6242938; doi:10.1038/s41598-018-34777-x)
Supplement: Supplementary file 2 — Clinical trial protocol [file 41598_2018_34777_MOESM2_ESM.doc]

**Entwicklung eines Präventionskonzeptes zur Förderung der Zahngesundheit**

**Development of a preventive concept to improve oral health**

Leiter der Studie

Head of Study OÄ Dr. med. dent. C. Frese

OÄ PD Dr. med. dent. D. Wolff

Poliklinik für Zahnerhaltungskunde

Klinik für Mund-, Zahn- und Kieferkrankheiten

Universitätsklinikum Heidelberg

Weitere an der Studie Beteiligte:

Further participants ZÄ S. Schick

ZÄ Th. Wohlrab

ZA F. Leciejewski

Study Nurse Maria Inceoglu

Verantwortliche Studienärzte

Principal investigators OÄ Dr. med. dent. C. Frese

OÄ Dr. med. dent. D. Wolff

Biometrikerin

Statistics Kathrin Stucke

Datum/Version des Untersuchungsplans

Date and version of the study plan 26.08.13 Version 1.3

**Zusammenfassung**

Obwohl das Versorgungsniveau im Bereich der Zahngesundheit in Deutschland in den letzten Jahrzehnten kontinuierlich gestiegen ist, bleiben Kariesentstehung und Kariesprävention ein zentrales Thema in der Zahnerhaltungskunde. Die Erfolge der präventiven Zahnheilkunde in Deutschland sind bei den Kindern und Jugendlichen durch die breite Verfügbarkeit von Fluoriden in der Gruppen- und Individualprophylaxe, sowie durch den Einsatz von Fissurenversiegelungen (Versiegelung der kariesgefährdeten Kauflächen durch eine dünne Kunststoffschicht) seit längerem nachgewiesen.

Nicht-kariesbedingte Zahnhartsubstanzläsionen resultieren dagegen oftmals aus kombinierten Effekten von Erosion, Abrasion und Attrition. Aufgrund einer gegenseitigen Verstärkung nehmen hier die Erosion und die Abrasion einen besonderen Stellenwert ein. Das „Erweichen“ und der unmittelbar folgende Abtrag von Zahnschmelz und Dentin erhöhen den Verlust von Zahnhartsubstanz dramatisch. Daten zur Prävalenz in der Bevölkerung zeigen, dass kariöse Läsionen und erosiver Zahnhartsubstanzverlust in jeder Altergruppe vorkommen [1]. Einzelne spezielle Risikogruppen wie zum Beispiel Ausdauersportler wurden im Vergleich zur allgemeinen Bevölkerung hinsichtlich der Prävalenz von Erosionen in unserer Arbeitsgruppe bereits untersucht. Das Hauptziel der Folgestudie ist die Ermittlung des präventiven Effekts von zwei speziell für den Erosionsschutz entwickelten Mundhygieneserie auf die Progredienz dentaler Erosionen und kariöser Läsionen bei Ausdauersportlern:

*Zinnchlorid und aminfluoridhaltige/natriumfluoridhaltige Mundspüllösung und Zahnpasta (elmex Erosionsschutz® Mundspüllösung und Zahnpasta, GABA GmbH, Lörrach, Germany).*

Bei der oben genannten Produktserie handelt es sich gemäß der Verordnung (EG) Nr.

1223/2009 des Europäischen Parlaments und des Rates vom 30. November 2009 um

kosmetische Mittel. Weitere Details zur Durchführung einer sonstigen Studie mit dieser

Produktserie sind der separaten Stellungnahme vom 26.08.2013 zu entnehmen.

Die Testgruppe besteht aus Amateuren, die Ausdauersport (vornehmlich Triathlon) ausüben. Innerhalb dieser Gruppe wird das Testprodukt an die Hälfte der Probanden ausgegeben, die instruiert werden und es zusätzlich zur häuslichen Mundhygiene verwenden sollen. Hierfür wird eine Randomisierung vorgenommen. Mit Hilfe dieser Daten soll es möglich sein, den präventiven Effekt des Testprodukts im Vergleich zur normalen häuslichen Mundhygiene bei Ausdauersportlern zu ermitteln. Außerdem ist es wünschenswert, bestimmte Risikogruppen für Karies und dentale Erosionen, wie beispielsweise Ausdauersportler, mit einem speziellen Präventionskonzept zur Förderung der Zahngesundheit zu unterstützen.

**Summary**

Although the level of care in the field of dental health in Germany has risen steadily in recent decades, caries and caries prevention remain a central theme in Dentistry. The success of preventive dentistry in Germany are among children and adolescents demonstrated by the wide availability of fluoride in the group and individual prophylaxis and the use of pit and fissure sealants (sealing of caries risk purchasing pools through a thin layer of plastic) for some time.

Non-carious tooth structure caused by lesions on the other hand often result from combined effects of erosion, abrasion and attrition. Due to a mutual reinforcement erosion and abrasion are of special value. The "softening" and the immediately following removal of tooth enamel and dentin increase the loss of tooth structure dramatically. Data on the prevalence in the general population show that caries lesions and erosive tooth loss occur in any age group. Individual special risk groups such as endurance athletes were examined in comparison to the general population with regard to the prevalence of erosions in our group already. The main objective of the follow-up study is to determine the preventive effect of commonly available special oral care products on the progression carious lesions and erosions in endurance athletes. The test group consists of amateurs, the endurance sports (mainly triathlon) exercise. Within this group, the test product is output to half of the subjects that will be using it in addition to oral hygiene and be instructed. For this purpose, randomization is performed. Using this data, it should be possible to determine the preventive effect of the test product as compared with normal oral hygiene in endurance athletes. Also, it is desirable that certain risk groups for dental caries and erosion, such as endurance athletes, to help with a special prevention program to promote dental health.

**Einleitung**

Aufgrund moderner Lebensgewohnheiten wie z. B. Diäten in vielfältiger Form, gepaart mit sportlicher Aktivität, treten spezielle Risikogruppen, wie beispielsweise Ausdauersportler in der Zahnheilkunde vermehrt in den Focus der Aufmerksamkeit. Zwar konnte in der vierten deutschen Mundgesundheitsstudie (DMS IV) der Bundeszahnärztekammer und der kassenzahnärztlichen Bundesvereinigung aus dem Jahr 2006 im Vergleich zur DMS III bei den Erwachsenen erstmals ein deutlicher Rückgang der kariesbedingten Zahnhartsubstanzdefekte nachgewiesen werden [2], dennoch gibt es eindeutige wissenschaftliche Hinweise, dass die Prävalenz der dentalen Erosionen in einzelnen Bevölkerungs- und Risikogruppen zunimmt. Die nicht-kariesbedingten Formen von Zahnhartsubstanzverlust werden selten in nationalen Querschnittsstudien zur Mundgesundheit untersucht und bleiben trotz steigender Prävalenz in diesen Kollektiven weitgehend unberücksichtigt. In Europa werden nur in Großbritannien im UK Childrens´ Dental Health Survey seit 1993 Daten zur Prävalenz von Erosionen erhoben. Der Vergleich der Daten aus dem Jahr 1993 und 1996/97 zeigt eine Zunahme der Prävalenz von Erosionen bei den 4-18 Jährigen [3, 4]. Eine in unserer Arbeitsgruppe bereits untersuchte Risikogruppe für die Entstehung dentaler Erosionen und kariöser Läsionen sind Ausdauersportler. Lange Trainingsphasen rufen im Körper diverse Effekte wie v. a. vermehrte Kapillarisierung, Zunahme der Mitochondrien, Verbesserung des aeroben Energiestoffwechsels und Ökonomisierung der Herzarbeit hervor [5]. In Bezug auf das stomatognathe System reduziert sich die Speichelfließrate und durch Mundatmung beim Training kommt es zur Austrocknung der Schleimhäute. Hinzu kommt der Konsum von Sportlergetränken und kohlenhydratreicher Sportlernahrung (z.B. Gele und Riegel), die erosives und kariogenes Potential aufweisen. Da Ausdauersportarten wie Triathlon und Marathon im Bereich des Freizeitsports immer beliebter werden, ist die Entwicklung präventiver Konzepte für diese Risikogruppen unerlässlich. Daher scheint die geplante Untersuchung der Wirksamkeit präventiver Produkte für die häusliche Mundhygiene sinnvoll und von hoher klinischer Relevanz.

**Introduction**

The introduction consists of general information and was therefore not translates into English language. Thank you for your understanding.

**Arbeitshypothese (anpassen)**

Die Arbeitshypothese ist, dass durch die instruierte Verwendung von zinnchloridaminfluorid- und natriumfluoridhaltiger Zahnpasta und Mundspüllösung die Ausprägung dentaler Erosionen und kariöser Läsionen in der Testgruppe

im Vergleich zur Kontrollgruppe beeinflusst werden kann.

Die Nullhypothese ist, dass zwischen Testgruppe und Kontrollgruppe kein Unterschied in

Bezug auf die Ausprägung dentaler Erosionen und kariöser Läsionen gefunden wird.

**Working hypothesis**

The general working hypothesis was that the manifestation of dental erosion in the intervention group changes over the observation period as compared to the control group. The special working hypothesis was defined as follows: The use of SnCl2/AmF/NaF-containing mouth rinsing solution and tooth paste leads to changes in the manifestation of dental erosion. The null hypothesis was that SnCl2/AmF/NaF-containing mouth rinsing solutions and tooth pastes have no effect on oral health.

**Ziele der Studie**

**Hauptzielkriterien:**

- Auftreten dentaler Erosionen (ja/nein)
- Auftreten kariöser Läsionen (ja/nein)
- Progredienz dentaler Erosionen (ja/nein)
- Progredienz kariöser Läsionen (ja/nein)
- Auftreten von Hypersensitivitäten (Graduierung mit Visual Analogue Scale (VAS))

**Nebenzielkriterien:**

- Orale Lokalisation dentaler Erosionen
- Orale Lokalisation kariöser Läsionen
- Speichelfließrate (stimuliert und unstimuliert in ml)
- Speichelpufferkapazität
- pH-Wert im Speichel

**Aims of the study**

**Primary endpoint**

- Presence of dental erosion (yes/no)
- Presence of carious lesions (yes/no)
- Progression of dental erosion (yes/no)
- Progression of carios lesions (yes/no)
- Presence of dentin hypersensitivity (yes/no)

**Secondary endpoint**

- Localization of dental erosion
- Localisation of carious lesions
- Saliva flow rate
- Buffering capacity of saliva
- Saliva pH

**Zur Gliederung der Studie**

Ausdauerathleten (vornehmlich Triathleten) aus der Region Heidelberg sollen zur Teilnahme an der Studie rekrutiert werden (geplante Fallzahl n=50). Innerhalb dieser Gruppe werden an die Teilnehmer randomisiert die Testprodukte zur zusätzlichen Verwendung bei der häuslichen Mundhygiene ausgegeben und die Probanden erhalten Instruktionen. Für alle Probanden, die sich mit einer Teilnahme an der Studie einverstanden erklärt haben, findet eine Eingangsuntersuchung in der Poliklinik für Zahnerhaltungskunde statt. Nach 6, 12, 18, 24, 30, 36, 42 und 48 Monaten werden Nachuntersuchen (Recalls) durchgeführt. Bei allen Untersuchungsterminen werden folgende Parameter erhoben:

1. Ergänzender Fragebogen zur allgemeinen und speziellen zahnärztlichen Anamnese (vor allem Graduierung Hypersensitivitäten auf VAS, Zufuhr von Getränken und Nahrungsmitteln, Art der Getränke und Nahrungsmittel während oder nach Ausdauerbelastung) (siehe Anhang 1)
2. Intraoraler Befund unter besonderer Berücksichtigung der erosiv und kariös betroffenen Zähne und des Schweregrads der dentalen Erosion (Lokalisation im Schmelz, Dentin, mit/ohne Pulpabeteiligung)
3. Erhebung des ICDAS II [7] (siehe Anhang 2)
4. Erhebung des BEWE-Score [6] (siehe Anhang 3)
5. Durchführung eines Speicheltests (GC Saliva Check BUFFER; GC, Leuven, Belgium) zu Bestimmung der
   1. Unstimulierten Speichelfließrate [ml]
   2. Stimulierten Speichelfließrate [ml]
   3. Pufferkapazität
   4. pH-Wert
6. Standardisierte intraorale Fotoaufnahmen
7. Dokumentationsmodelle (Abformungen Ober- und Unterkiefer)

Die Speichelproben der Probanden werden für die oben genannten Analysen bei -25 Grad eingefroren.

**Work flow of the study:**

The participants in the intervention group were instructed to use a stannous fluoride-containingmouth rinse and a toothpaste containing for daily oral hygiene at home.

The participants in the control group did not get any products, but were instructed to use fluoridated toothpaste (1500 ppm) along with the conventional oral hygiene products they were using at home. They were explicitly told not to use oral hygiene products containing stannous fluoride.

Participants were recalled every six months: after 6, 12, 18, 24, 30, 36, 42 und 48 months. Each study appointment included:

1. Detailed anamnesis (see attachment 1)
2. Intraorale examination with special regard to dental erosion and caries prevalence
3. ICDAS II [7] (siehe Attachment 2)
4. BEWE-Score [6] (siehe Attachment 3)
5. Saliva test (GC Saliva Check BUFFER; GC, Leuven, Belgium) zu Bestimmung der
   1. Unstimulated saliva flow rate [ml]
   2. Stimulated saliva flow rate [ml]
   3. Buffering capacity
   4. pH value
6. Standardised intraoral photographs
7. Cast models (upper and lower jaw)

The saliva samples were stored for further in-vitro analyses at -25 degrees.

**Wirkungen (therapeutisch, diagnostisch):** nicht vorhanden und nicht erwartet

**Effect (therapeutic and diagnostic):** none

**Unerwünschte Wirkungen, sonstige Risiken, Belastungen für den Studienteilnehmer:** Für die Studienteilnehmer entsteht für die Eingangsuntersuchung bzw. die jeweilige Recalluntersuchung und die Durchführung der Fotos, Abformungen und des Speicheltests eine zeitliche Belastung von etwa 60 Minuten. Weitere Risiken, unerwünschte Wirkungen oder Belastungen liegen nicht vor. Es werden keine zusätzlichen invasiven

und/oder belastenden Untersuchungen vorgenommen.

**Unfavourable Events, Risk for the participants:**

For the participants of this study the time required for the baseline examination and the follow up appointments is 60 minutes. Further risks or unfavourable events are not to be expected. No addition al invasive treatments are planned.

**Studiendesign(-typ):**

Monozentrische, prospektive Fallkontrollstudie

**Studydesign:**

Moncantric, prospective case controll study

**Randomisierungsverfahren:**

Die Zuteilung der Testprodukte an die Probanden erfolgt mittels einer Randomisierungsliste, die vom Institut für Medizinische Biometrie und Informatik erstellt wird. Als Verfahren wird eine Blockrandomisierung gewählt.

**Randomisation:**

They were randomized by block randomization (sequentially numbered envelopes) into intervention and control group. The random allocation sequence was generated by the statistician at Institut für Medizinische Biometrie und Informatik; enrolment and assignment of participants to interventions was done by the principal investigator.

**Einschlusskriterien:**

- Probanden sind älter als 18 Jahre
- Die Einverständniserklärung liegt vor
- Probanden verfügen über ausreichende Deutschkenntnisse
- Probanden erfüllen ein wöchentliches Trainingspensum von mind. 5 Stunden
- Probanden sind Ausdauersportler
- Probanden haben keine körperlichen Einschränkungen oder Behinderungen, die eine normale Mundhygiene nicht ermöglichen

**Inclusion criteria**

- Gender: Both, male and female
- Minimum Age: 18 Years
- Maximum Age: 64 Years
- subjects older than 18 years
- Written informed consent
- subjects have sufficient knowledge of German
- volunteers fulfill a weekly training schedule of at least 5 hours
- subjects are endurance athletes
- subjects have no physical limitations or disabilities that do not allow normal oral hygiene

**Ausschlusskriterien:**

- Jugendliche unter 18 Jahren
- Keine Einwilligung zur Teilnahme an Studie
- Keine ausreichenden Deutschkenntnisse
- Probanden erfüllen ein wöchentliches Trainingspensum unter 5 Stunden
- Probanden sind keine Ausdauersportler
- Zahnärztliches Personal oder Zahnmedizinstudenten
- Körperliche Einschränkungen oder Behinderungen, die dem/r Proband/in eine normale Mundhygiene nicht ermöglichen
- Zeichen von stark vernachlässigter Mundhygiene (schwere und akute parodontale Erkrankung oder schnell fortschreitende Karies)
- Probandin ist schwanger oder stillt

**Exclusion criteria**

- Adolescents under 18 years
- No written informed consent to participate in study
- Lack of sufficient knowledge of German language
- volunteers fulfill a weekly training schedule under 5 hours
- subjects are no endurance athletes
- dental staff or dental students
- Physical limitations or disabilities that do not allow the subject to a normal oral hygiene
- strong sign of neglected oral hygiene (severe periodontal disease and acute or rapidly progressing caries)
- test subject is pregnant or breastfeeding

**Begleittherapie (soweit einschlägig):** erlaubt

**Additional therapy:** permitted

**Abbruchkriterien:**

Individuelle Abbruchkriterien: Proband möchte nicht mehr an der Studie teilnehmen

Abbruchkriterien für die Gesamtstudie: Nicht bekannt

**Termination of study:** Individual termination of study, participant does not want to participate anymore.

**Statistisches Design:**

Die vorliegende Untersuchung wird als Fallkontrollstudie durchgeführt und dient der Hypothesengenerierung. Die Festlegung der Fallzahl basiert daher auf der Abschätzung der Anzahl an Ausdauerathleten, die einer Studienteilnahme einwilligen werden. Es wird erwartet, dass etwa 50 Ausdauerathleten aus der Region um Heidelberg bereit sein werden, an der Studie teilzunehmen. Die Zahl der zu untersuchenden Personen wird somit aus Gründen der Machbarkeit auf 50 festgelegt.

Alle erhobenen Daten werden mit Hilfe deskriptiver Statistik analysiert (Angabe von absoluten und relativen Häufigkeiten bzw. Mittelwert, Standardabweichung, Median, Interquartilabstand (IQR), Konfidenzintervalle). Die Untersuchung eines möglichen Unterschiedes zwischen den beiden Gruppen in Bezug auf den primären Endpunkt, das Auftreten dentaler Erosionen und kariöser Läsionen, erfolgt mit dem Chi-Quadrat-Test. Unter Berücksichtigung von potentiellen Einflussfaktoren auf die Zielgröße wird eine logistische Regression durchgeführt. Außerdem sollen verschiedene Paarvergleiche zwischen den Gruppen erfolgen. Dabei werden kontinuierliche Merkmale mittels T-Test oder U-Test und kategoriale Daten mittels Chi²-Test verglichen. Die erhaltenen p-Werte werden dabei rein deskriptive interpretiert und haben keinen konfirmatorischen Wert. Fehlende Werte werden nicht imputiert. Die Auswertung erfolgt mit SAS for Windows, Version 9.1.

**Statistics**

Descriptive analyses were conducted by the information gathered from the patient and the clinical investigations of the baseline examination. Mean, SD, median, minimum and maximum were determined for continuous variables while absolute and relative frequencies were computed for categorical variables, alongside descriptive p-values to assess the homogeneity between treatment groups (using Wilcoxon-Mann-Whitney tests for continuous and chi-squared tests for categorical variables). Statistical analysis of the primary endpoint “dental erosion” served to detect group differences. In doing so, account was taken of the long-term course of the study, and resulting measurement repetitions were taken into consideration. The primary analysis of the BEWE was done using a linear mixed model for repeated measurements (MMRM) adjusting for saliva pH value, BEWE at baseline using an unstructured covariance matrix.

Boxplots and confidence intervals were plotted to illustrate the treatment effect over time.

All analyses were done using the software package SAS® System 9.4 (SAS Inc., Cary/NC, USA). RStudio Desktop 1.1.383 was used to create the graphics.

**Ethische und rechtliche Aspekte**

**Ethical aspects, declaration of Helsinki:** It was assumed that the content of declaration of Helsinki is well known. Therefore it was not translated into English language.

- Die Untersuchung wird in Übereinstimmung mit der Deklaration von Helsinki und der Berufsordnung für Ärztinnen und Ärzte der Landesärztekammer Baden-Württemberg in den jeweils aktuellen Fassungen durchgeführt.
- Die Teilnahme der Patienten/Probanden an der Untersuchung ist freiwillig.
- Die Zustimmung kann jederzeit, ohne Angabe von Gründen und ohne Nachteile für die weitere medizinische Versorgung, zurückgezogen werden.
- Die Patienten/Probanden werden vor Studienbeginn schriftlich und mündlich über Wesen und Tragweite der geplanten Untersuchung, insbesondere über den möglichen Nutzen und eventuelle Risiken, aufgeklärt. Ihre Zustimmung wird durch Unterschrift auf der Einwilligungserklärung dokumentiert.
- Bei Rücktritt von der Studie wird bereits gewonnenes (Daten-) Material vernichtet oder beim Proband angefragt, ob er mit der Auswertung des Materials einverstanden ist (nur bei Pseudonymisierung möglich).
- Der Studienplan wird vor Studienbeginn der Ethikkommission der Medizinischen Fakultät Heidelberg zur Begutachtung vorgelegt. Es wird nicht mit dem Einschluss von Probanden begonnen, bevor nicht das schriftliche, zustimmende Votum der Ethikkommission vorliegt.
- Die Namen der Probanden und alle anderen vertraulichen Informationen unterliegen der ärztlichen Schweigepflicht und den Bestimmungen des Bundesdatenschutzgesetzes (BDSG).
- Eine Weitergabe von Patienten-/Probandendaten erfolgt ggf. nur in pseudonymisierter Form. Dritte erhalten keinen Einblick in Originalkrankenunterlagen

**Unterschriften**

**Signature**


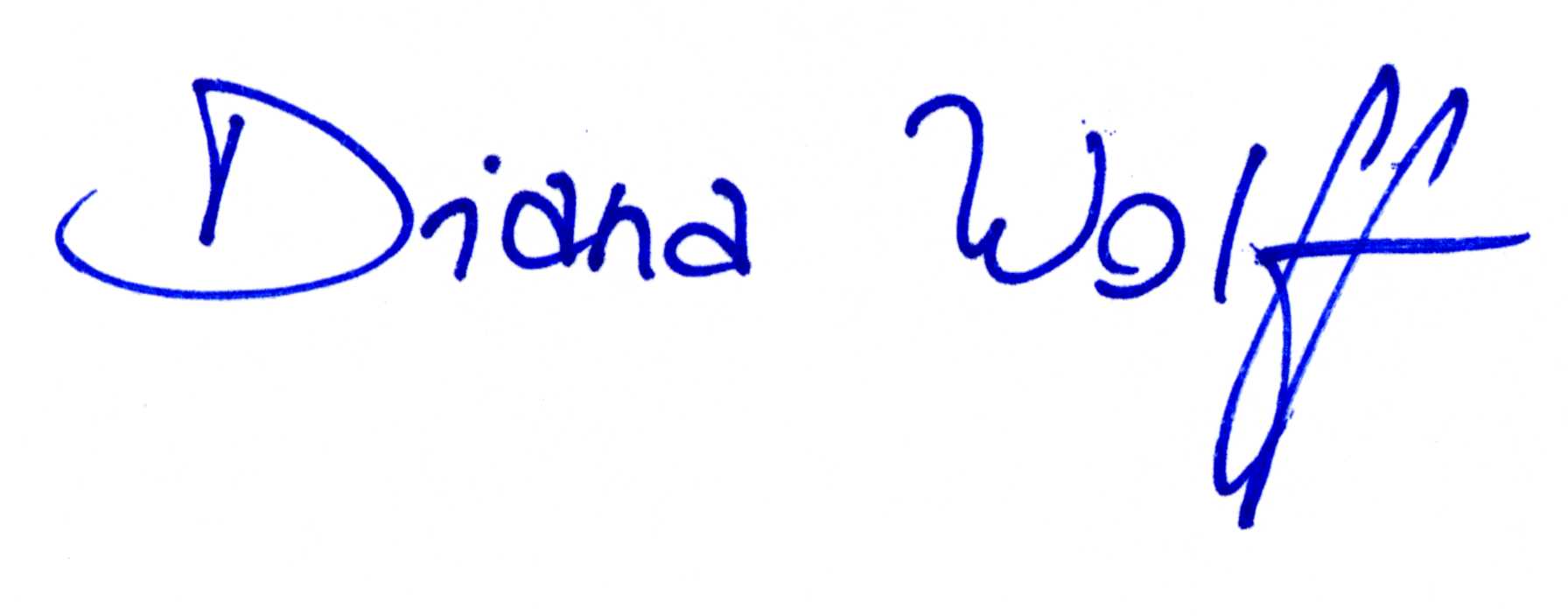

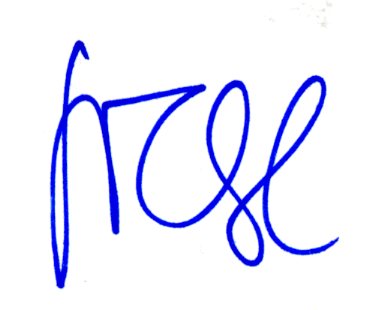


Dr. C. Frese Dr. D. Wolff

**Literatur**

[1] Lussi A (ed.): Dental erosion- From Diagnosis to Therapy

*Monographs in Oral Science, Editor: G.M. Whitford, vol. 20, Karger, Basel (2006)*

[2] Vierte Deutsche Mundgesungheitsstudie (DMS IV), *Herausgeber: Kassenzahnärztliche Bundesvereinigung und Bundeszahnärztekammer (2006)*

[3] Nunn JH, Gordon PH, Morris AJ, Pine CM, Walker A: Dental erosion- changing prevalence? A review of British National childrens´survey. *Int J of Peadiat Dent; 13 (2):98-105 (2003)*

[4] Lussi A, Hellwig E, Ganss C, Jaeggi T: Buonocore Memorial lecture. Dental erosion

*Oper dent; 34(3):251-62 (2009)*

[5] Rost R (ed): Lehrbuch der Sportmedizin

*Deutscher Ärzteverlag, Köln (2001)*

[6] Bartlett D, Ganss C, Lussi A.: Basic Erosive Wear Examination (BEWE): a new scoring system for scientific and clinical needs.

*Clin Oral Investig. Mar; 12 Suppl 1:S65-8 (2008)*

[7] Nyvad B, Machiulskiene V, Baelum V. Reliability of a new caries diagnostic system differentiating between active and inactive caries lesions. Caries Res 1999;33:252-60.

**Anhang 1 : Detaillierter Fragebogen zur Anamnese**

**Ergänzender Fragebogen zur Anamnese**

**Entwicklung eines präventiven Konzepts zur Förderung der Zahngesundheit bei Ausdauersportlern**

Alter: _________________

Geschlecht: männlich O weiblich O

Beruf: _________________

Sportart: _________________

Größe: _________________

Gewicht: _________________

Seit wie vielen Jahren betreiben Sie Ausdauersport? Seit ________ Jahren

Training/Woche (in h) gesamt: _________________

Laufen:___________

Radfahren:________

Schwimmen:_______

Ernährung während des

Trainings: __________________________________________

Getränke während des

Trainings: __________________________________________

Wieviel wird getrunken (pro Stunde Training)? _________________________

Woraus wird getrunken? __________________________________________

Spezielle Diät: ja O nein O

Wenn ja, welche:____________________________

Wie schmerzempfindlich (heiß/kalt) sind Ihre Zahnhälse (bitte ankreuzen)?

1 (gar nicht) 2 3 4 5 6 7 8 9 10 (sehr)

Wie häufig/lange putzen Sie Ihre Zähne?

_______________________________________________________________

Welche Hilfsmittel (z.B. Zahnseide) verwenden Sie?

_______________________________________________________________

Welche Zahnpasta verwenden Sie?

_______________________________________________________________

Verwenden Sie Mundspüllösungen? Wenn ja, welche?

_______________________________________________________________

Haben Sie das Gefühl, das Sie mit den Zähnen knirschen oder pressen während des Ausdauertrainings?

ja O nein O **VIELEN DANK!**
